# Reducing Test Anxiety by Device-Guided Breathing: A Pilot Study

**DOI:** 10.3389/fpsyg.2022.678098

**Published:** 2022-05-23

**Authors:** Zehava Ovadia-Blechman, Ricardo Tarrasch, Maria Velicki, Hila Chalutz Ben-Gal

**Affiliations:** ^1^School of Medical Engineering, Afeka—Tel Aviv Academic College of Engineering, Tel Aviv, Israel; ^2^School of Education, Tel Aviv University, Tel Aviv, Israel; ^3^Sagol School of Neuroscience, Tel Aviv University, Tel Aviv, Israel; ^4^School of Industrial Engineering and Management, Afeka—Tel Aviv Academic College of Engineering, Tel Aviv, Israel

**Keywords:** test anxiety, respiration, device-guided breathing, pilot study, self-treatment

## Abstract

Test anxiety remains a challenge for students and has considerable physiological and psychological impacts. The routine practice of slow, Device-Guided Breathing (DGB) is a major component of behavioral treatments for anxiety conditions. This paper addresses the effectiveness of using DGB as a self-treatment clinical tool for test anxiety reduction. This pilot study sample included 21 healthy men and women, all college students, between the ages of 20 and 30. Participants were randomly assigned to two groups: DGB practice (*n* = 10) and wait-list control (*n* = 11). At the beginning and the end of 3-weeks DGB training, participants underwent a stress test, followed by measures of blood pressure and reported anxiety. Anxiety reduction in the DGB group as compared to controls was not statistically significant, but showed a large effect size. Accordingly, the clinical outcomes suggested that daily practice of DGB may lead to reduced anxiety. We assume that such reduction may lead to improved test performance. Our results suggest an alternative treatment for test anxiety that may also be relevant for general anxiety, which is likely to increase due to the ongoing COVID-19 pandemic.

## Highlights

- Recent decrease in mental and psychosocial well-being may raise anxiety levels.
- Device-guided breathing may aid as a treatment for anxiety.
- Students trained in device-guided breathing showed significant anxiety reduction.

## Introduction

Tests are an important part of any student’s educational career. Students who suffer from anxiety during exams have difficulty expressing their abilities optimally, which can lead to frustration and impair their learning. Test anxiety is a combination of physiological over-arousal, tension, and somatic symptoms, along with worry, dread, fear of failure, and catastrophizing, which occur before or during test situations (Zeidner, 1998). Technological changes and environmental factors such as a general decrease in an individual’s quality of life may affect test anxiety levels (Alyahya and Almutairi, 2019; Chalutz Ben-Gal, 2020). Several test anxiety treatments have been assessed and reviewed, using various performance outcomes (Tryon, 1980). However, despite advances in test anxiety prevention and management, the worldwide prevalence of newly-diagnosed individuals with anxiety in general, and test anxiety in particular, is expected to rise in the near future due to the COVID-19 pandemic outbreak in December 2019 (Fessell and Cherniss, 2020; World Health Organization, 2020). Furthermore, it has been shown that distance learners are especially prone to mental disorders in general and, more specifically to stress (Harrer et al., 2019).

In recent years, there has been an increase in the implementation of anxiety reduction measures by means of psychological, social, cognitive and educational interventions within educational institutions (Van Dinther et al., 2011; Yeager and Walton, 2011). These interventions also include contemplative practices such as yoga, journaling (Ergas, 2014), and mindfulness (Praissman, 2008; Tarrasch, 2015; Garrison, 2017). Such programs help in supporting students with a variety of challenges as for example learning disabilities and test anxiety (Vaughn et al., 2015; Mitchell and Gansemer-Topf, 2016; von der Embse et al., 2018; Alyahya and Almutairi, 2019). A recent study among higher education students compared two non-pharmacological interventions: mindfulness and device-guided slow breathing, and found that both interventions led to stress reduction (Gabriely et al., 2020). In this paper, we refer to Device-Guided Breathing (DGB), as an exercise that aims at lowering the respiratory frequency to less than 10 breaths per minute with prolonged exhalation guided by an electronic device called RESPeRATE™ (2breathe Technologies Ltd., Israel). This device was cleared by the FDA as an over-the-counter device for self-treatment of hypertension and stress (more details are given below). A growing body of research has emerged over the last few decades examining the effect of DGB on various physiological and psychological symptoms. Several studies have shown that DGB usage decreases general anxiety levels (Gavish, 2010; Ovadia-Blechman et al., 2017) and anxiety experienced by individuals in medical settings (Morarend et al., 2011) and various clinical heart conditions (Lachowska et al., 2019b). In addition, it also aids in post-traumatic stress disorder (PTSD) symptoms (Fonkoue et al., 2018), and in reducing elevated sympathetic nervous activity (Oneda et al., 2010; Harada et al., 2014; Ovadia-Blechman et al., 2017; Fonkoue et al., 2018), and in sensitizing the baroreflex function, which enhances the blood pressure control (Fonkoue et al., 2018). Recent evidence supports the view that slow DGB improves cardiovascular reactivity to mental stress and health-related quality of life in heart failure patients (Lachowska et al., 2019a,b). Deep slow breathing eventually leads to a reduction in cortisol release. In detail, the mechanism underlying cortisol reduction is that deep slow breathing leads to pulmonary stretch receptors activity (Russo et al., 2017), which in turn may cause an increase in heart rate during inspiration and decrease during expiration. This change affects the baroreflex and chemoreceptors, that in turn modulate epinephrine release and the HPA-axis and modulate corticotropic-releasing hormone, which mediates release of adrenocorticotropic hormone (ACTH). Finally, ACTH reduction reduces release of cortisol from the adrenal cortex, thus reducing stress (Dancy and Kim, 2018). It has been shown that reductions in salivary cortisol are also associated with mood improvement (Cruess et al., 2000).

However, despite this evidence, the effects of DGB on students experiencing test anxiety (which impacts test performance) have not yet been evaluated. Accordingly, the objective of the present study was to evaluate the effect of DGB on anxiety levels of students experiencing test anxiety.

### Test Anxiety

Test anxiety is a psychological condition in which people experience extreme levels of anxiety and discomfort during the course of a test or prior to taking it (Zeidner, 2010). While many people experience some degree of anxiety in general and, more specifically, test anxiety during various testing scenarios, test anxiety can practically impair and interfere with any learning process. It may lead to poor levels of test performance as well as additional malfunctioning (Zeidner, 2010; von der Embse et al., 2018).

Previous studies on test anxiety discussed its theoretical aspects and conceptualization and its influences on test performance. For example, Hembree (1988, 1990) investigated test anxiety within educational settings and found that it is characterized by context-specific stimuli (e.g., classroom instruction) and academic subject specific reactions (e.g., math anxiety). A recent meta-analytic study on test anxiety examined the influence of test anxiety on a variety of educational outcomes by analyzing 238 studies performed between the years 1988 and 2018. The study concluded that test anxiety significantly impeded a wide range of educational performance outcomes, including standardized tests, university entrance exams, and grade point average (von der Embse et al., 2018). The emotional aspect of test anxiety is expressed by emotional arousal, nervousness and fear. It may sometimes be accompanied by physiological symptoms, including heart palpitations and shortness of breath (Michael, 2014).

The severity of test anxiety varies considerably among individuals. Minor symptoms may include light levels of anxiety, which may take the form of a slight stomach pain, while more severe symptoms may include concentration difficulties, test attention malfunctions and longer-term symptoms (Michael, 2014). Some patients may experience rising heartbeat levels, shakiness as well as helplessness. In some severe documented cases, patients may feel nausea, shortness of breath, or even a full-scale panic attack (Michael, 2014).

Causes of test anxiety may stem from the interaction between external environmental factors and internal personality-related factors (Hembree, 1988). Examples of environment-related factors include demographic variables such as level of education, economic status and cultural background (von der Embse et al., 2018). Notwithstanding, the internal personality-related factors associated with test anxiety may include personality traits such as perfectionism, fear of failure, low self-esteem levels, and low self-confidence levels (von der Embse et al., 2018). Moreover, research shows that deficiencies or lack of study capabilities, negative past learning experiences, and excessive pressure levels posed by external environmental factors may all increase test anxiety levels (Zeidner, 2010).

The effects of anxiety on physiological variables have been investigated over the years (Albright et al., 1991; Hering et al., 2013). Previous studies indicated that anxiety management procedures incorporating relaxation techniques have the potential to lower cardiovascular responses to anxiety. The most likely reason for the changes observed is a general decrease in sympathetic nervous system activity (Albright et al., 1991; Hering et al., 2013).

As mentioned above, several test anxiety treatment tools and techniques have been assessed. For example, desirable changes in lifestyle, such as exercise and proper nutrition, may reduce anxiety (Martinsen and Raglin, 2007). Anxiety-relief relaxation techniques and programs such as yoga and mindfulness interventions have also been shown to be effective (Chung et al., 2012; Gabriely et al., 2020; Querstret et al., 2020). Additional tools include music therapy (Scheufele, 2000) combined with bio-feedback using electroencephalography (EEG; Aalbers et al., 2017; de Zambotti et al., 2019). However the effects of a respiratory training device, on test anxiety have not yet been analyzed (Dong et al., 2010).

The implications of the COVID-19 world pandemic on test anxiety are yet to be determined, however, in line with recent calls from the World Health Organization (WHO), it is plausible to assume that this global crisis generates anxiety in a similar way to other stressful situations, among various populations (World Health Organization, 2020). Indeed, recent studies suggest a direct effect on anxiety, among various populations (Asmundson et al., 2020; Iimura, 2022). Such anxiety may rise even more in the near future due to the expected decrease in mental and psychosocial well-being caused by the outbreak (Chen et al., 2020; Fessell and Cherniss, 2020; Wang et al., 2020).

### Test Anxiety Treatment Based on Device-Guided Breathing

Previous studies assessing the positive effects of slow breathing have shown benefits among several clinical measures (Olsén et al., 2015), including cardiorespiratory functions (Ankad Roopa et al., 2011), emotion regulation (Arch and Craske, 2006), motor skills (Yadav and Mutha, 2016), relaxed resting state, and joy (Philippot et al., 2002).

The DGB used in this study guides its user gradually and interactively from spontaneous breathing to slow breathing at five to six breaths per minute with prolonged exhalation and without the need for conscious effort (Gavish, 2010; Ovadia-Blechman et al., 2017). The device monitors respiration with a belt-type sensor placed on the upper abdomen or chest; analyzes inspiration and expiration durations in real-time, and composes musical tones in real-time with durations that correspond to the monitored inhale and exhale movements but with slightly longer durations, according to proprietary algorithms. The “breathing guiding” function is achieved when the user synchronizes breathing movements with these tones, which creates a closed-loop operation, hereafter called “device-guided breathing” (Gavish, 2019).

Studies indicate that the ability to noninvasively monitor changes in skin microcirculation reflects local and systemic changes as well as improves both diagnosis and treatment of certain diseases (Ovadia-Blechman et al., 2015, 2016, 2018; Lustig et al., 2018). Moreover, a recent study sheds light on a new measurement trend in respiration—vasomotion (spontaneous oscillations in the tone of blood-vessel walls, independent of heartbeat, innervation, or respiration), coupled with the application of DGB treatment, which may characterize microcirculation response at tissue oxygenation below a measurable threshold (Ovadia-Blechman et al., 2017). These findings may serve as an explanation for the beneficial effects of DGB on anxiety treatment.

Known side effects, together with the cost of antihypertensive drugs, have stimulated the search for non-pharmacological approaches to control test anxiety and blood pressure (BP), both as first-line and as adjunctive treatments (Bandelow et al., 2015). Some studies investigated the influence of using DGB as a clinical therapy for the treatment of hyperventilation syndrome. These devices were found to be efficient for the purpose of anxiety reduction and served as a relief factor from the hyperventilation syndrome (Grossman et al., 1985). Additional studies have utilized DGB in assisting patients who suffer from PTSD symptoms (Fonkoue et al., 2018), psychological symptoms such as sleeping disorders, cognitive disorders, and high blood pressure (Gavish, 2019).

Previous studies on the negative effects of test anxiety on the one hand, along with the positive outcomes of DGB usage on the other, led to the present study, which, to the best of our knowledge, explores for the first time the effectiveness of DGB in reducing test anxiety.

## Materials and Methods

### Participants

Twenty-one healthy men (68%) and women (32%), all college students, between the ages of 20 and 30, participated in this study. Students were recruited from Afeka College through the Dean of Students Office’s website. Participants were randomly assigned to two groups: DGB practice (*n* = 10) and waiting-list control (*n* = 11). All procedures performed in this study were in accordance with and received the approval of the Tel-Aviv University Ethics Committee (January 2017). Written informed consent was obtained from all participants, who formally declared that to the best of their knowledge they are generally healthy. Exclusion criteria included: high blood pressure, cardiac problems, asthma, and recent symptoms of cold or flu.

### DGB Practice

**Figure 1:**
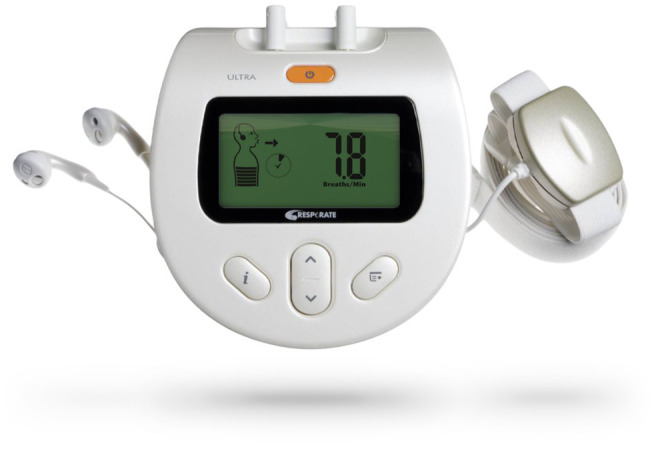
The RESPeRATE device used for device-guided breathing intervention (with the permission of the manufacturer).

Students received the RESPeRATE device (RESPeRATE Inc., United States) for daily practice at home, 15 min per day, for 3 weeks (Figure 1). The RESPeRATE device guides the user to shift from a spontaneous to a slow breathing rate below 10 breaths/min (Schein et al., 2001; Gavish, 2010; Ovadia-Blechman et al., 2017). The device includes a belt-type breathing sensor placed on the upper abdomen or chest connected to a computerized box. The sensor monitors the breathing pattern *via* the variations of the abdomen torso circumference. The electronic box determines from the signal, in real-time, the duration of inhalation and exhalation for each breath. The computerized box includes a breath-guiding melody that comprises one tone for inhalation and another for exhalation, with which the user is requested to synchronize breathing movements. Since the tones’ duration is slightly longer than that of the monitored inhale/exhale movements, the device gradually guides the user interactively from a spontaneous breathing rate (typically 15 breaths/min) to about five to six breaths per minute without a conscious effort and with a relatively prolonged exhalation (Gavish, 2010; Ovadia-Blechman et al., 2017). All participants in the treatment group, reported daily practice according to the guidelines, throughout the study.

The compliance of users with the DGB exercise, following merely written instructions for use was evaluated in a multi-center study, in which the exercise performance was documented by an internal memory installed in the device. Results demonstrated that 95% of those who operated the device at least once achieved slow breathing using the written instructions provided (Elliott et al., 2004). The performance of DGB exercise was further validated, as a part of a physiological study with a student population from the present college (Ovadia-Blechman et al., 2016).

### Test Anxiety

Measuring anxiety levels in general, and test anxiety more specifically, is challenging (Hembree, 1988). The challenges of measuring anxiety originate from three factors: (1) the difficulty of collecting physiological data in a real-time stressful environment, (2) the variability between subjects with regards to anxiety, and (3) the difficulty of estimating the level of anxiety (Hembree, 1988). Aiming to overcome these limitations, some scholars consider laboratory-based experiments for assessing anxiety, rather than performing real-time measurements. Previous studies operationalized anxiety level by measuring, within the laboratory environment, the effects of various anxiety-inducing stimuli on several physiological signals (Hembree, 1988).

The mental arithmetic anxiety test, requiring subjects to perform a series of arithmetic operations, is frequently used for inducing anxiety. It is considered an accurate tool for assessing test performance, compared to other methods (Ushiyama et al., 1991). It is simple to administer and it does not require instruments. The test includes seven subtractions from 700 as quickly and as accurately as possible, for a period of two and a half minutes.

To assess anxiety immediately following the stressor, participants completed the state scale of the State–Trait Anxiety Inventory. The scale has good validity and reliability properties *α* = 0.90 (Spielberger et al., 1970).

### Protocol

**Figure 2:**
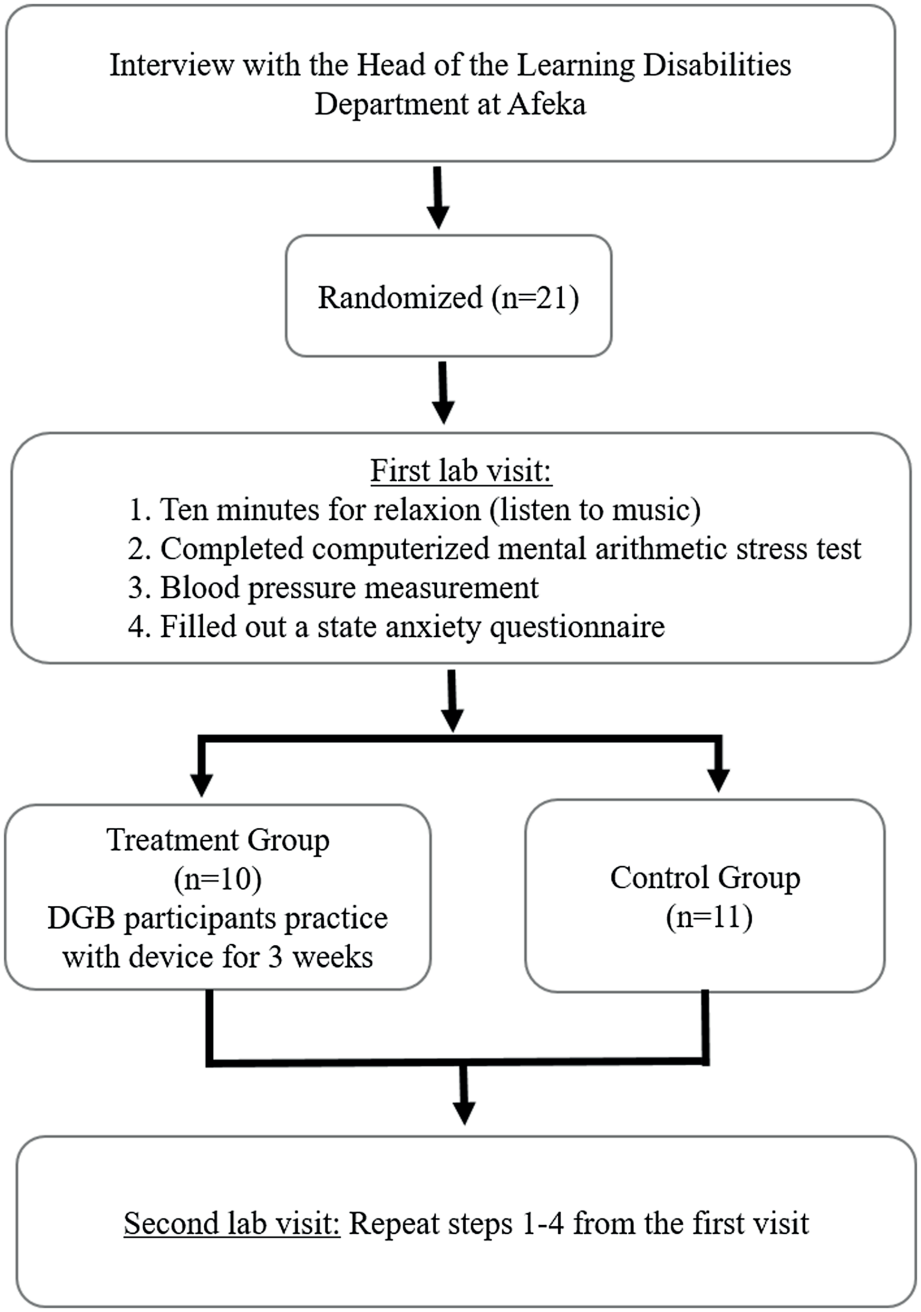
Experimental protocol.

The experimental protocol included four steps, as shown in Figure 2. Candidates were recruited to the study based on self-report of experiencing learning difficulties. A pre-screening unstructured interview was performed by an expert in learning difficulties, in order to subjectively evaluate test anxiety. Participants were then randomly assigned to the treatment and control groups. Both groups were required to visit the lab twice. During each of the two sessions, throughout the first 10 min, participants were asked to sit and relax while reading or listening to music. This selection was in accord with the 1995-US guidelines that required at least 5 min of rest (Canzanello and Sheps, 1998).

Thereafter, they performed a computerized mental arithmetic stress test for two and a half minutes. Immediately after completion of the computerized mental arithmetic stress test, blood pressure was measured. Both brachial systolic blood pressure (SBP) and diastolic blood pressure (DBP) were measured using a standard digital blood pressure monitor (OMRON IC, IntelliSense TM, Japan). At the end of each session, participants filled out a state anxiety questionnaire (STAI questionnaire, as detailed below).

The time between the two visits was 3 weeks. During this time, participants in the treatment group performed daily home practice using a DGB, while the control group did not. The students were taught how to use the device at home. The second visit coincided with the beginning of the exams period. The selection of 3-weeks treatment period was based on previous findings that the main reduction of high blood pressure in response to daily use of the device occurred within 3 weeks (Grossman et al., 2001; Meles et al., 2004).

### Data Analysis

Since the two groups differed in their baseline anxiety measure (*t*-test, *p* = 0.006), percent change was calculated for each participant as the difference between the post-measure and baseline, divided by the baseline, for the anxiety measure, SBP and DBP. *T*-test for independent samples was used to assess the difference between the groups (device-guided breathing vs. control) in anxiety, SBP and DBP percent change. Analyses were performed using SYSTAT 12 (SYSTAT Software Inc., San Jose, California, United States).

## Results

**Figure 3:**
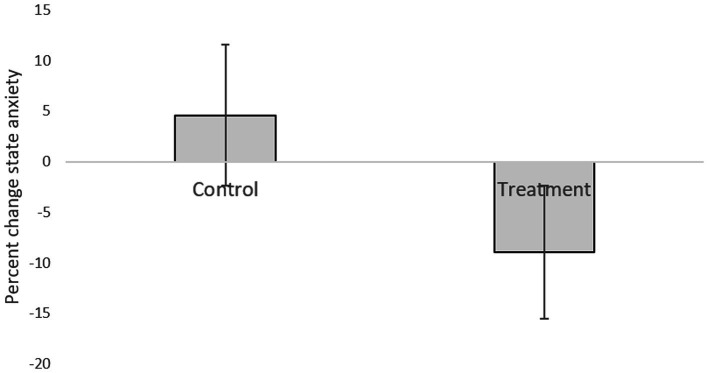
Anxiety level difference between the two lab visits in both the treatment and control groups.

**Figure 4:**
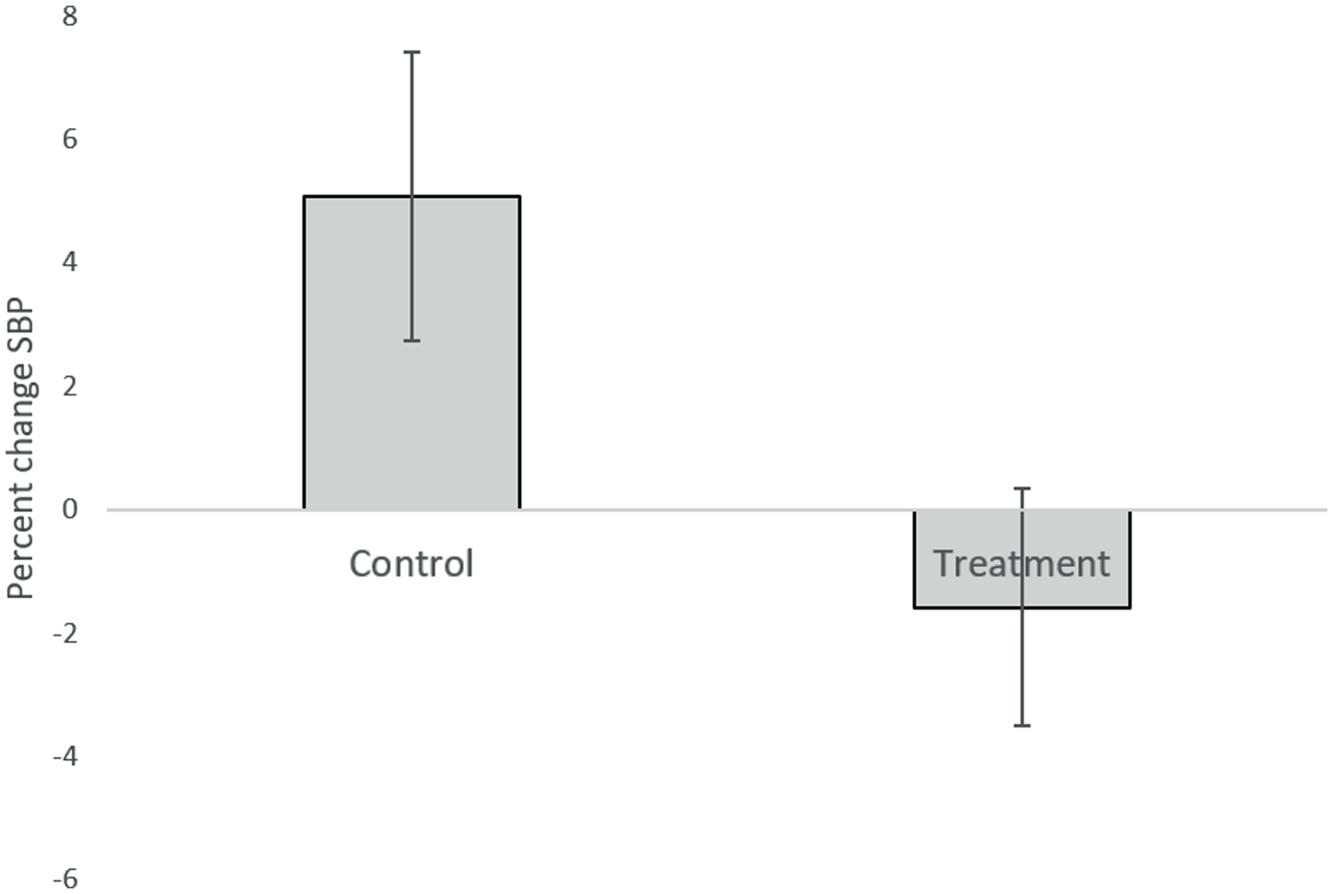
Systolic blood pressure difference between the two lab visits in both the treatment and control groups.

Table 1 presents descriptive statistics of the study variables at pre and post 3 weeks of daily practice with device-guided breathing, separately for control and the experimental participants. Our main hypothesis concerning a larger reduction in anxiety in the device-guided breathing group (Figure 3), was non-significant [*t*(19) = 1.40, *p* = 0.18, *Cohen’s d* = 0.61], however the effect size was of a medium size. SBP percent change significantly differed between the groups [*t*(19) = 2.18, *p* = 0.04, Cohen’s *d* = 0.96], with a reduction in the experimental group, compared to an increase in the control group (Figure 4). DBP percent change did not significantly differ between the groups [*t*(19) = 0.90, *p* = 0.38, *Cohen’s d* = 0.40].

**Table 1:** Means and standard deviations of SBP [mmHg], DBP [mmHg], and anxiety, pre and post 3 weeks of daily practice with DGB, separately for control and the experimental participants.

	Control				DGB			
	Pre		Post		Pre		Post	
	Mean	SD	Mean	SD	Mean	SD	Mean	SD
SBP	115.8	11.705	121	7.894	118.6	12.644	116.4	10.83
DBP	71.86	8.4501	73.32	4.966	77.95	10.101	76.95	8.516
Anxiety	35.36	5.334	36.36	6.64	46.5	10.448	42.3	12.68

## Discussion

The purpose of this pilot study was to assess the effect of DGB on the anxiety levels of patients experiencing test anxiety. Our results indicate that daily practice using device-guided breathing may lead to reduced test anxiety. Although the reduction in anxiety in the DGB group was not significant (perhaps due to the small sample), the effect size was medium, suggesting a possible effect.

High test-anxious individuals display an increased sympathetic nervous system activity (Bian et al., 2022). The physiological mechanisms that can explain the beneficial effects of slow breathing or DGB on this condition are likely to be associated with reducing this elevated sympathetic activity as follows: the need to maintain oxygen supply requires that under isocapnic conditions slow breathing lead to greater lung inflation. Moderate lung inflation was demonstrated to almost completely inhibit muscle sympathetic neural activity from late inspiration to mid-expiration in normal subjects (Seals et al., 1990, 1993). Greater lung inflation that accompanies DGB, or during slow/deep breathing enhances the suppression of the neural sympathetic activity (Harada et al., 2014). The effect stems from activation of low-threshold pulmonary stretch receptors located in the lung and chest wall that initiate vagal afferents (Shepherd, 1981). This process leads to vasodilation in a number of vascular territories such as intact limb, skin, muscle, kidney and splanchnic vascular bed (Daly, 1995), resulting in lowering resistance to blood flow and acute reduction in blood pressure (Parati et al., 2007). Furthermore, local tissue perfusion is provided by microvessels (arterioles) that establish the peripheral resistance to blood flow, where the vessel’s diameter is determined by the tone of smooth muscles embedded in the arteriolar wall that is controlled by sympathetic neural activity (Burton, 1965). Arterioles display spontaneous and continuous diameter oscillations at about 6 cycles/min called “vasomotion” that plays an important role in controlling peripheral resistance (Intaglietta, 1990). Slow breathing at 5–6 breaths/min couples with vasomotion (*via* the respiration-driven oscillation in the sympathetic neural activity), which results in increased oscillations at the capillary blood flow in a resonance-like form that was observed during DGB (Ovadia-Blechman et al., 2017). A related phenomenon may be the resonance-like increase in the heart rate variability by slow breathing at a rate of 6 breaths/min called “resonance breathing,” which synchronizes increase/decrease in heart rate with the inhalation/exhalation, that are likely to couple with blood pressure oscillation *via* the baroreflex function (Vaschillo et al., 1983, 2002, 2006; Lehrer et al., 1997). “Prolonged exhalation,” as induced by DGB, reduces the chance of inspiratory muscle fatigue, since its perfusion occurs mainly (or solely) during exhalation. Inspiratory muscle fatigue leads to sympathetic activation (Roussos and Macklem, 1982).

According to previous studies, anxiety in general, and more specifically test anxiety are mental situations, which are accompanied by a wide range of psychological and physiological symptoms (Zeidner, 2010). The physiological symptoms are related to changes in central nervous system activity (CNS). The central nervous system is so named because it integrates information it receives, and coordinates and influences the activity of all parts of the body. Anxiety affects sympathetic nervous system activity and accordingly body functioning, diminishing a person’s ability to function effectively and efficiently (Khan et al., 2017).

Previous studies that investigated the influence of DGB as a clinical therapy for the treatment of the hyperventilation syndrome found reductions in anxiety that served as a relief factor from hyperventilation syndrome (Grossman et al., 1985). Another study (case report) supported the view that DGB decreases “fight-flight” reactions (de Jong and Boersma, 2010).

Research suggests that a slow and regular breathing pattern has a number of beneficial effects on the reflex control of the cardiovascular system (Gavish, 2010). Ten minutes of daily use of the DBG elicited a clinically significant reduction in the BP level in patients with hypertension (Grossman et al., 2001; Schein et al., 2001). Additional research based on DGB suggests that it may be useful as an adjunctive treatment for BP control (Sharma et al., 2011). In the current study, SBP percent change significantly differed between the groups. This finding stems from an increased SBP in the control group. This may be explained by increased stress during the study exams period. It may be possible that the DGB training prevented the increase in pressure observed in the control group (Figure 4). This explanation is supported by previous studies, mentioned above, which have shown the effect of DGB practice on reduction of blood pressure.

Notwithstanding, some studies addressed the challenges of using DGB as a self-treatment tool in the home setting. It was found that the relationship between breathing pattern and BP levels changed over the treatment period, indicating treatment effectiveness. Moreover, the treatment device successfully managed to reduce participants’ breathing rate effortlessly and prolong exhalation (Gavish, 2010), which leads to relaxation.

We believe that our findings may add additional information to both policymakers and students. Recently, scholars highlighted several interventions, with both social and behavioral ingredients, aimed at preventing and treating the indirect effects of the COVID-19 pandemic (Eaton and Kalichman, 2020). Thus, we view the pandemic as a potential trigger to consider DGB practice in educational settings after replicating the present results using larger randomized controlled trials. As a result of the COVID-19 pandemic, education has partly shifted to remote learning and is more technology-based. This form of learning may increase general as well as test anxiety levels among students (Clabaugh et al., 2021). We believe that our results, which implemented individual self-regulation of test anxiety, may have the potential to support possible amelioration in other domains such as quality of life (Chalutz Ben-Gal, 2020). Moreover, students and test-takers may benefit by successfully mastering test management, especially during stressful exam periods, as currently experienced. The adoption process can be implemented by preparation and practice in advance. DGB adoption has a great potential to aid due to its ease of administration, safety, portability, convenience, and non-invasive nature.

## Conclusion

Device-guided breathing was found to be a potential tool for test-anxiety reduction. If confirmed in further studies, our results may suggest the adoption of DGB as an alternative treatment for test anxiety. We assume that such adoption may lead to improved test performance. Furthermore, DGB may also be relevant for general anxiety reduction, which is likely to increase due to the ongoing COVID-19 pandemic.

## Strengths and Limitations

This study utilized a device-guided breathing medical device approved by the FDA and associated with no side effects. It offers an easy and enjoyable self-treatment tool. This may be specifically useful for higher education students who nowadays perform most of their studies remotely, challenged by the ongoing COVID-19 implications.

This study has some limitations. First, our sample was extremely small. Second, our results are based on an intentional clinical trial in the laboratory. As such, they may not indicate students’ actual and real-time performance indicators (e.g., final exam grade). Third, the second measure in our study was concomitant with the beginning of the exams period, and accordingly our results may have been affected by this fact. Accordingly, there is a need to further examine the clinical results on a larger sample, utilizing actual testing settings. Finally, we acknowledge that our main significant finding stems from physiological changes, rather than from actual shifts in anxiety levels in a real-life situation.

## Future Research

This research may be further developed through several avenues. First, we believe that the results of this study may be further assessed in accordance with the identified challenges resulting from the ongoing COVID-19 pandemic. The World Health Organization, as well as other public health authorities in the world, are acting to contain the pandemic. However, this time of crisis generates stress throughout the population. Key influences include mental health and psychosocial well-being in various target groups, including students who are actively engaged in learning (Fessell and Cherniss, 2020; World Health Organization, 2020). In line with WHO recommendations, there is a growing need to find opportunities to amplify positive and hopeful stories, as well as positive images of the future.

Second, several biological and psychological variables may be also collected and analyzed within various populations; for example, in work and organization-related contexts, where employees experience high levels of work stress (Lucini et al., 2007; Fessell and Cherniss, 2020). Third, an interesting line of research may be to examine test anxiety, together with physiological and psychological measures, to assess other interventions, such as mindfulness and relaxing activities (e.g., yoga), or examine the effect on students with learning disabilities and/or attention deficit hyperactivity disorders (Gabriely et al., 2020; Gál et al., 2021).

Finally, it would be interesting to analyze device-guided breathing utilization effects on quality-of-life parameters (Chalutz Ben-Gal, 2020) in the digital and technological era, which is still evolving.

## Data Availability Statement

The raw data supporting the conclusions of this article will be made available by the authors, without undue reservation.

## Ethics Statement

The study was reviewed and approved by Tel-Aviv University Ethics Committee. Participants provided their written informed consent to participate in this study.

## Author Contributions

ZO-B: conceived the study and designed the trial, supervision, data analysis and data interpretation, writing—original draft, and writing—review and editing. RT: data analysis and data interpretation, writing—original draft, and writing—review and editing. MV: data presentation, writing—original draft, and writing—review and editing. HC: data interpretation, writing—original draft, and writing—review and editing. All authors contributed to the article and approved the submitted version.

## Conflict of Interest

The authors declare that the research was conducted in the absence of any commercial or financial relationships that could be construed as a potential conflict of interest.

## Publisher’s Note

All claims expressed in this article are solely those of the authors and do not necessarily represent those of their affiliated organizations, or those of the publisher, the editors and the reviewers. Any product that may be evaluated in this article, or claim that may be made by its manufacturer, is not guaranteed or endorsed by the publisher.
